# Association between prehospital ROX index with 30-day mortality among septic shock

**DOI:** 10.1186/s40001-024-01902-8

**Published:** 2024-05-31

**Authors:** Romain Jouffroy, Tristan Fabre, Basile Gilbert, Stéphane Travers, Emmanuel Bloch-Laine, Patrick Ecollan, Josiane Boularan, Vincent Bounes, Benoît Vivien, Papa Gueye

**Affiliations:** 1 Intensive Care Unit, Ambroise Paré Hospital, Assistance Publique - Hôpitaux de Paris, 9 avenue Charles De Gaulle, 92100 Boulogne-Billancourt, France; 2 U1018 INSERM, Centre de Recherche en Epidémiologie et Santé des Populations, Paris Saclay University, Gif-sur-Yvette, France; 3 EA 7329, Institut de Recherche Médicale et d’Épidémiologie du Sport, Institut National du Sport, de l’Expertise et de la Performance, Paris, France; 4 SAMU 972, Centre Hospitalier Universitaire de Martinique, Fort-de-France Martinique, France; 5 EA 7525, University of the Antilles, French West Indies, France; 6 Department of Emergency Medicine, SAMU 31, University Hospital of Toulouse, Toulouse, France; 7 Paris Fire Brigade, Paris, France; 8 Emergency Department, Cochin Hospital, Paris, France; 9 Emergency Department, SMUR, Hôtel Dieu Hospital, Paris, France; 10 Intensive Care Unit, SMUR, Pitie Salpêtriere Hospital, 47 Boulevard de l’Hôpital, 75013 Paris, France; 11 SAMU 31, Castres Hospital, Castres, France; 12 SAMU de Paris, Service d’Anesthésie Réanimation, Hôpital Universitaire Necker - Enfants Malades, Assistance Publique - Hôpitaux de Paris, Université Paris Cité, Paris, France; 13 UR5_3 PC2E Pathologie Cardiaque, Toxicité Environnementale et Envenimations, Université des Antilles, Pointe-à-Pitre, France

**Keywords:** Septic shock, Prehospital setting, Mortality, ROX index

## Abstract

**Purpose:**

Respiratory dysfunction is one of the most frequent symptoms observed during sepsis reflecting hypoxemia and/or acidosis that may be assessed by the ROX index (ratio of oxygen saturation by pulse oximetry/fraction of inspired oxygen to respiratory rate). This study aimed to describe the relationship between the prehospital ROX index and 30-day mortality rate among septic shock patients cared for in the prehospital setting by a mobile intensive care unit (MICU).

**Methods:**

From May 2016 to December 2021, 530 septic shock patients cared for by a prehospital MICU were retrospectively analysed. Initial ROX index value was calculated at the first contact with MICU. A Cox regression analysis after propensity score matching was performed to assess the relationship between 30-day mortality rate and a ROX index ≤ 10.

**Results:**

Pulmonary, digestive and urinary sepsis were suspected among 43%, 25% and 17% patients, respectively. The 30-day overall mortality reached 31%. Cox regression analysis showed a significant association between 30-day mortality and a ROX index ≤ 10: adjusted hazard ratio of 1.54 [1.08–2.31], *p* < 0.05.

**Conclusions:**

During the prehospital stage of septic shock patients cared for by a MICU, ROX index is significantly associated with 30-day mortality. A prehospital ROX ≤ 10 value is associated with a 1.5-fold 30-day mortality rate increase. Prospective studies are needed to confirm the ability of prehospital ROX to predict sepsis outcome since the prehospital setting.

## Background

Every year sepsis concern more than 50 million people worldwide despite research performed during the last 40 years. Sepsis still remains a major health problem [1–3] with an increasing incidence and high morbidity and mortality despite of recent advances in its management [1, 4–9]. Every year nearly 11 million deaths worldwide are due to sepsis [3]. The overall sepsis mortality rate still reaches 30% at 28 days but is higher, i.e., 50% for the most severe form of sepsis, i.e., septic shock [10, 11].

Since 2017, the World Health Assembly and the World Health Organization adopted resolutions to improve, prevent, diagnose, and sepsis management [12] to reduce health impact of sepsis.

Sepsis recognition and severity assessment are mainly based on clinical judgement and scoring [13]. When the resources are scarce, e.g., in the extra hospital setting, the initial sepsis diagnose is often difficult while it is widely admitted that early detection and treatment instauration improve patient outcome [14–16]. Due to the lack of a clinical sign specificity for sepsis, scores and indexes were developed and are widely used to help or guide physicians in the daily bedside decision-making process.

Sepsis may lead to multi-organ dysfunction including cardiovascular, respiratory, renal, neurological, hematological, and hepatic dysfunctions. Independently of its origin, sepsis induces a metabolic acidosis caused by renal injury and tissue hypoperfusion and/or hypoxemia related to organs dysfunction [17–19]. The respiratory dysfunction is one of the most frequent observed during sepsis and septic shock. Hypoxemia and/or acidosis induce as respiratory rate increase and partial pressure of oxygen (PaO_2_/FiO_2_) decrease. Beyond these pathophysiological considerations, in and out of hospital epidemiological studies report sepsis mainly comes from respiratory (50%) and digestive (25%) and less frequently from urinary tract (5%) [20–22].

The clinical usefulness of the ROX index, oxygen saturation divided by the inspired oxygen concentration (FiO_2_), and then by the respiratory rate, was described first in 2016 [23] among patients suffering from pulmonary disease and confirmed by other studies [24, 25]. Recently, Lee et al. reported that the ROX index was lower in non-survivors with a ROX index cutoff less than or equal to 10 suggesting that the ROX index could be used as a prognostic marker in sepsis among adult patients admitted to the emergency department with a sepsis or septic shock diagnosis [26].

This retrospective study aims to describe the relationship between prehospital ROX index and 30-day mortality rate among septic shock patients cared for in prehospital setting by a mobile intensive care unit (MICU).

## Methods

### Patients

As previously reported [27], in France prehospital emergency medical service is named SAMU (Urgent Medical Aid Service). SAMU is a phone call centre responding to the patients’ complaints [28] to determine the best care pathway. For life-threatening emergencies, a mobile intensive care unit (MICU) may be dispatched to the scene [29].

From May 2016 to December 2021, prehospital septic shock patients according to the 2012 sepsis-2 conference criteria [30] extrinsically applied by the MICU physicians of 9 French hospital centres (Necker-Enfants malades Hospital, Lariboisière Hospital, La Pitié Salpêtrière Hospital, Hotel Dieu Hospital, APHP, Paris—France; The Paris Fire Brigade Paris—France; The Toulouse University Health Centre, Toulouse—France and the Castres Hospital, Castres—France), were retrospectively analyzed. Patients younger than 18 years, and/or pregnant, and/or with serious comorbid conditions with an unknown prehospital life support and/or with guardianship or curatorship were not included in the dataset [31]. The operative sepsis-2 definition considering a septic shock a condition of refractory hypotension despite vascular filling or normotension with hypoperfusion signs was chosen because prehospital lactatemia assessment is not possible in all French MICU.

Patients’ demographic characteristics, presumed prehospital origin of sepsis, the first recorded MICU contact prehospital and the last prehospital vital sign values [systolic blood pressure (SBP), diastolic blood pressure (DBP) and mean arterial pressure (MAP)] were collected for the dataset. Heart rate (HR), pulse oximetry (SpO2), respiratory rate (RR), body core temperature, and Glasgow coma scale (GCS), plasma blood glucose concentration, duration of prehospital care, and prehospital treatments delivered (ABT type and dose, fluid volume expansion type and dose, as well as catecholamine type and dose, mechanical ventilation) collected for the dataset. Comorbidities reflecting the underlying condition [32] were also reported: hypertension, coronary heart disease, chronic cardiac failure, chronic renal failure, chronic obstructive pulmonary disease, history of cancer, diabetes mellitus were also collected to take into account. Body mass index (BMI) was calculated by dividing weight (kg) by [height (m) * height (m)].

Length of stay (LOS) in the ICU, in-hospital LOS and 30-day mortality were retrieved from medical reports in case of in-hospital death or by call when the patient was discharged from the hospital. The Simplified Acute Physiology Score (SAPS-2) was calculated 24 h after ICU admission [33].

The ROX index was calculated by dividing the initial values, e.g., at the first MICU contact of prehospital patient’s pulse oximetry prior any oxygen supplementation, by the inspired oxygen concentration (FiO_2_), and then by the RR [23].

To minimize data abstraction bias [34], the data collection was performed by a single investigator (RJ) using a standardized abstraction template established prior the study. To minimise transcription errors, two investigators (TF and PG) re-check the data and identified no error.

### Ethical considerations

The study was approved by the French Society of Anaesthesia and Intensive Care ethics committee on December 12th, 2017 (Ref number: IRB 00010254-2017-026). According to the French law, this non-interventional retrospective observational study the ethical committee waived consent of patients.

### Statistical analysis

Results are expressed as mean with standard deviation and interquartile range [Q1–Q3], and as absolute value and percentage depending on the type of variable.

ROX index was analyzed as a continuous variable and as qualitative variable using a threshold of ROX ≤ 10 for abnormal value according to Lee et al. study reporting that a ROX index ≤ 10 is an independent prognostic factor for 28-day mortality in patients with sepsis or septic shock admitted to the emergency department [26] suggesting that ROX index could be useful for sepsis prognostication.

To reduce the effect of confounders on 30-day mortality and on ROX calculation, a propensity score matching was performed to balance the differences in baseline characteristics between patients with prehospital ROX ≤ 10 and those with prehospital ROX > 10 [35]. The propensity score was estimated using logistic regression based on potential confounders on 30-day mortality and on ROX calculation: age, chronic cardiac failure, chronic renal failure, chronic obstructive pulmonary disease, coronary heart disease, BMI, history of cancer, diabetes mellitus, SAPS-2, prehospital fluid expansion [31] and prehospital antibiotic therapy (ABT) administration [9]. The nearest neighbour matching method was used to match patients based on the logit of the propensity score [35]. The balance of covariates after matching was assessed by absolute mean differences with a threshold of 10% [36].

Imbalance matching was assessed with standardized mean deviation. Baseline characteristics were compared between cases and controls by paired tests in the matched sample.

In the propensity score-matched cohort, a survival analysis using Cox proportional hazard regression was used to compare 30-day mortality rate according to a prehospital ROX ≤ 10 and a prehospital ROX > 10. Proportional hazards assumption was verified for each Cox model variable by Kaplan–Meier curves and the log-rank test. Results are expressed by an adjusted Hazard ratio (HRa) with 95 percent confidence intervals [95 CI].

All tests were two-sided with a statistically significant* p* value of < 0 0.05. All analyses were performed using R 3.4.2 (http://www.R-project.org; the R Foundation for Statistical Computing, Vienna, Austria).

## Results

### Patient characteristics

Five-hundred and thirty septic shock patients among which 341 patients were male gender (64%) with a mean age of 69 ± 15 years cared for by a MICU were retrospectively analyzed. The mean SAPS-2 score was 60 ± 21. The median length of stay in a hospital was 10 [5–18] days and the ICU length of stay was 4 [2–8] days (Table 1).

**Table 1:** Population characteristics

	Overall population (*n* = 530)	ROX ≤ 10 (*n* = 117)	ROX > 10 (*n* = 413)	*p* value
Demographics				
Age (years)	69 ± 15	72 ± 16	69 ± 14	0.158
Weight (kg)	74 ± 20	70 ± 20	74 ± 20	0.141
Height (cm)	170 ± 12	170 ± 10	169 ± 12	0.632
BMI (kg m^−2^)	27.8 ± 37.5	24.1 ± 6.1	28.7 ± 4.3	0.120
Comorbidities				
Coronary heart disease	104 (20%)	15 (13%)	89 (22%)	0.558
Chronic cardiac failure	134 (25%)	21 (18%)	113 (27%)	0.527
Chronic renal failure	75 (14%)	8 (6%)	67 (16%)	0.456
COPD	186 (35%)	11 (9%)	68 (16%)	0.806
Cancer history	79 (13%)	27 (23%)	159 (38%)	0.679
Diabetes mellitus	151 (28%)	24 (21%)	51 (12%)	0.557
Prehospital initial values				
Initial SBP (mmHg)	97 ± 30	**94 ± 29**	**103 ± 33**	**0.027**
Initial DBP (mmHg)	58 ± 19	**57 ± 20**	**60 ± 22**	0.262
Initial MAP (mmHg)	71 ± 22	**83 ± 22**	**76 ± 26**	**0.016**
Initial HR (beats min^−1^)	114 ± 28	117 ± 28	69 ± 21	**< 10**^**–3**^
Initial RR (movements min^−1^)	30 [22–36]	44 [40–48]	29 [20–32]	**< 10**^**–3**^
Initial pulse oximetry (%)	92 [85–96]	80 [72–88]	93 [89–97]	**< 10**^**–3**^
Initial body core temperature (°C)	38.3 [36.5–39.1]	38.6 [36.5–39.4]	38.3 [36.4–39.1]	0.872
Initial Glasgow coma scale	**14 [12–15]**	**13 [9–15]**	**14 [13–15]**	**0.007**
Initial blood lactate (mmol L^−1^)	5.8 ± 3.4	6.1 ± 3.7	5.8 ± 3.3	0.529
Fluid expansion (mL)	750 [500–1000	750 [500–500]	1000 [500–1250]	0.158
Fluid expansion/body weight (mL kg^−1^)	11 [7–18]	11 [7–17]	12 [8–18]	0.497
Norepinephrine administration	155 (29%)	30 (26%)	125 (30%)	0.111
Norepinephrine dose (mg h^−1^)	1.0 [0.5–2.0]	1.5 [0.2–2.0]	1.2 [0.5–2.0]	0.854
Prehospital ABT administration	132 (25%)	23 (20%)	109 (26%)	0.515
Prehospital duration (min)	71 ± 34	78 ± 42	75 ± 32	0.376
Prehospital final values				
Final SBP (mmHg)	106 ± 25	104 ± 25	108 ± 25	0.181
Final DBP (mmHg)	62 ± 18	63 ± 20	63 ± 18	0.812
Final MAP (mmHg)	77 ± 19	76 ± 21	78 ± 19	0.290
Final HR (beats min^−1^)	107 ± 25	119 ± 23	106 ± 25	**< 10**^**–3**^
Final RR (movements min^−1^)	25 [19–30]	35 [30–39]	24 [18–30]	**< 10**^**–3**^
Final pulse oximetry (%)	97 [94–99]	96 [92–97]	97 [95–99]	**< 10**^**–3**^
Final body core temperature (°C)	38.0 [36.0–39.0]	38.1 [36.1–39.0]	38.2 [37.4–39.8]	0.873
Final Glasgow coma scale	**13 [11–15]**	**13 [10–15]**	**14 [12–15]**	**0.004**
Final blood lactate (mmol L^−1^)	4.2 ± 3.3	4.8 ± 3.1	4.1 ± 3.2	0.139
Prehospital ROX	**15.81 ± 5.94**	**8.39 ± 1.14**	**17.20 ± 5.42**	–
Prehospital mechanical ventilation	46 (9%)	18 (15%)	28 (7%)	0.633
Hospital parameters				
SAPS-2 score	60 ± 21	62 ± 20	60 ± 21	0.315
In-ICU length of stay (days)	4 [2–8]	7 [2–16]	4 [2–8]	**0.001**
In-hospital length of stay (days)	10 [5–18]	15 [6–27]	10 [5–17]	**0.013**

Results are expressed as mean and standard deviation for quantitative parameters (normal distribution), as median and interquartile range for quantitative parameters (non-Gaussian distribution) and as an absolute value and percentage for qualitative parameters

*BMI* body mass index, *SBP* systolic blood pressure, *DBP* diastolic blood pressure, *MAP* mean arterial pressure, *HR* heart rate, *RR* respiratory rate, *ICU* intensive care unit, *SAPS-2* simplified acute physiology score 2nd version, *COPD* chronic obstructive pulmonary disease, *ABT* antibiotic therapy, *min* minutes, *LOS* length of stay

Values in bold indicate a *p* value < 0.05 between prehospital ROX ≤ 10 group and prehospital ROX > 10 group

*P* value corresponds to the comparison between patients with prehospital ROX ≤ 10 and prehospital ROX > 10

Pulmonary, digestive and urinary infections were suspected in 43%, 25% and 17% of the cases, respectively (Table 2).

**Table 2:** Presumed septic shock origins

Origin	*n* (percentage)
Pulmonary	230 (43%)
Digestive	130 (25%)
Urinary	88 (17%)
Cutaneous	33 (6%)
Meningeal	11 (2%)
Gynaecological	3 (0.5%)
Ear nose throat	2 (0.5%)
Cardiac	2 (0.5)
Unknown	31 (6%)

Data are expressed in absolute value and the corresponding percentages are indicated into brackets (due to rounding percentage sum exceeds 100%)

The 30-day overall mortality rate reached 31% (165 patients).

Among the 132 patients (25%) who received prehospital ABT, 98 patients (74%) received a 3rd generation cephalosporin (40% cefotaxime and 60% ceftriaxone) without any reported adverse event related to prehospital ABT administration.

All patients received crystalloids infusion for prehospital hemodynamic optimization with a median fluid expansion volume of 750 [500–1000] ml. Norepinephrine infusion was delivered to 155 patients (29%) with a median dose of 1.0 [0.5–2.0] mg h^−1^ (Table 1).

### Main measurement

In the overall population, the mean initial ROX was 15.81 ± 5.94 and 117 patients (22%) had a prehospital ROX ≤ 10. Table 1 reports the comparison between patients with prehospital ROX ≤ 10 and prehospital ROX > 10 before propensity score matching.

**Fig. 1:**
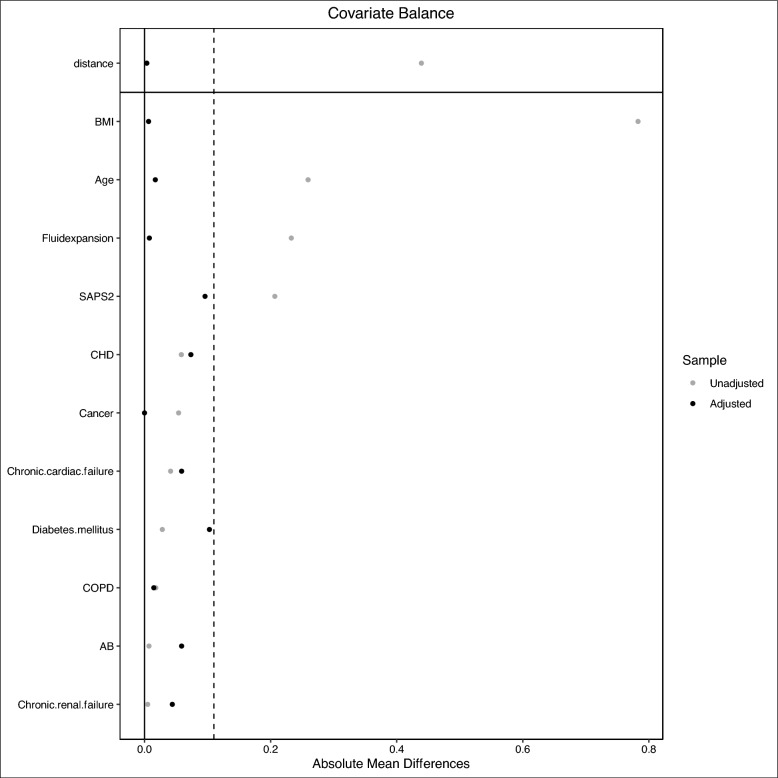
Absolute mean differences between patients with prehospital ROX ≤ 10 and prehospital ROX > 10 after matching

After propensity score matching for prehospital ROX ≤ 10, 68 patients with a prehospital ROX ≤ 10 were compared with 57 patients with a prehospital ROX > 10. Comparisons are reported in Table 3 and the absolute mean differences between subgroups after propensity score matching are depicted in Fig. 1.

**Table 3:** Cox regression analysis results

Covariate	HR [95 CI]	*p* value
Prehospital ROX ≤ 10	1.11 [1.05–1.17]	***0.001***
Age	1.02 [0.99–1.05]	0.136
Chronic cardiac failure	2.40 [0.96–4.17]	0.058
Chronic renal failure	1.85 [0.71–4.79]	0.207
COPD	1.43 [0.57–3.59]	0.444
Coronary heart disease	0.96 [0.43–2.15]	0.928
BMI	1.01 [0.94–1.09]	0.704
Cancer	1.69 [0.91–3.13]	0.098
Diabetes mellitus	0.47 [0.22–1.01]	0.052
SAPS-2	1.03 [0.99–1.05]	0.061
Prehospital fluid expansion	1.00 [0.99–1.01]	0.268
Prehospital ABT therapy	0.58 [0.26–1.27]	0.017

Results are expressed by hazard ratio (HR) with 95 percent confidence interval [95 CI]

*HR* hazard ratio, *95 CI* 95 percent confidence interval, *BMI* body mass index, *COPD* chronic obstructive pulmonary disease, *ABT* antibiotic therapy, *SAPS-2* simplified acute physiology score 2nd version

Using Cox regression analysis on matched population, we observed a significant association between 30-day mortality and prehospital ROX ≤ 10 with an aHR of 1.54 [1.08–2.31] (*p* < 0.05). Cox regression analysis results are summarized in Table 4.

**Table 4:** Comparison of predictive variable for 30-day mortality included in the propensity score before and after matching

Prehospital ROX ≤ 10	Before matching *n* = 530			After matching *n* = 125		
PS covariate	Cases	Controls	*p* value (d*)	Cases	Controls	*p* value (d*)
	*n* = 117	*n* = 413		*n* = 68	*n* = 57	
Age	72 ± 16	69 ± 14	< 10^–3^	72 ± 15	71 ± 13	0.655
Chronic cardiac failure	21 (18%)	113 (27%)	0.051	17 (25%)	15 (26%)	0.603
Chronic renal failure	8 (6%)	67 (16%)	0.090	7 (10%)	4 (7%)	0.522
COPD	11 (9%)	68 (16%)	0.182	11 (16%)	8 (14%)	0.740
Coronary heart disease	15 (13%)	89 (22%)	0.083	14 (21%)	7 (12%)	0.220
BMI	24.1 ± 6.1	28.7 ± 4.3	0.019	24.3 ± 6.1	24.4 ± 4.6	0.862
Cancer	27 (23%)	159 (38%)	0.364	23 (34%)	20 (35%)	0.882
Diabetes mellitus	24 (21%)	51 (12%)	0.379	21 (31%)	23 (40%)	0.271
SAPS-2	62 ± 20	60 ± 21	< 10^–3^	63 ± 19	60 ± 20	0.333
Fluid expansion	750 [500–500]	1000 [500–1250]	< 10^–3^	750 [500–1000]	1000 [500–1200]	0.758
ABT therapy	23 (20%)	109 (26%)	0.246	23 (34%)	18 (32%)	0.364
30-day mortality rate	50 (43%)	120 (29%)	< 10^–3^	38 (67%)	30 (44%)	< 10^–3^

Values are expressed as mean ± SD or number (%). *d* corresponds to the standard mean deviation value

*PS* propensity score, *LOS* length of stay, *COPD* chronic obstructive pulmonary disease, *ABT* antibiotic therapy, *SAPS-2* simplified acute physiology score 2nd version

**Fig. 2:**
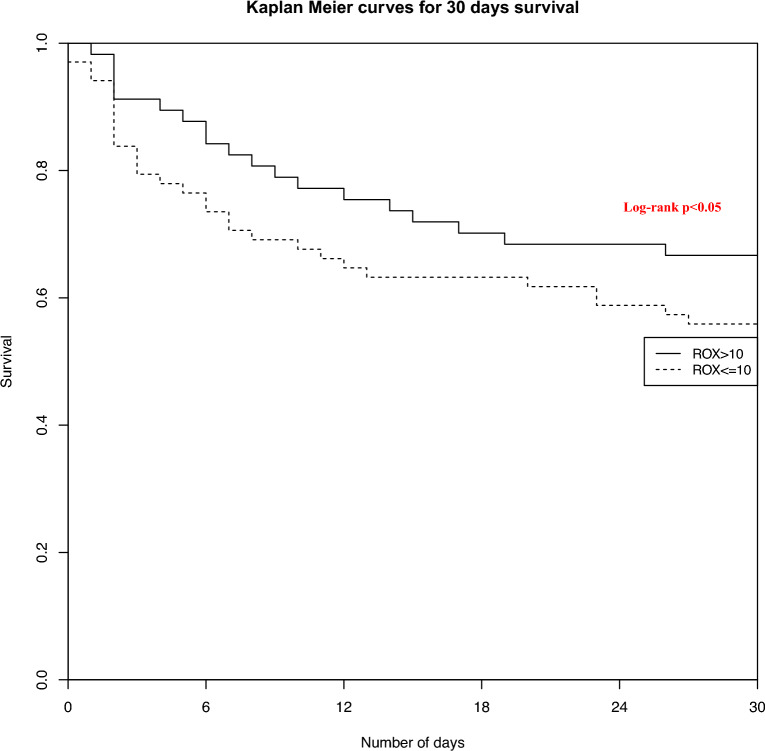
Kaplan–Meier curves of 30-day survival between prehospital ROX ≤ 10 and prehospital ROX > 10 after matching

Figure 2 depicts Kaplan–Meier curves after confounder adjustment for 30-day survival between prehospital ROX > 10 and prehospital ROX ≤ 10 after matching (Fig. 2).

## Discussion

In this study, we report a significant association between 30-day mortality and prehospital ROX index. An initial prehospital ROX index < 10 is associated with a 1.5-fold 30-day mortality increase among septic shock patients cared for by a MICU in the prehospital setting.

Early identification of septic patients at risk of poorer evolution and an high mortality is crucial because they are those who most need and most benefit from an early, aggressive therapeutic management, one of the key-element allowing sepsis mortality decrease [14].

Excluding shock, for sepsis severity assessment, clinical signs may be not sufficient because of their lack sensitivity and specificity. Consequently, to try to fill this gap, scoring was developed [13]. Initial scoring was based on clinical signs and thereafter, biological elements were added to improve performances. The most-known scores for sepsis are SOFA and SAPS-2 scores [33, 37], both developed and validated in the ICU and in the ED. However, both, SOFA and SAPS-2, because requiring biological results needing few hours to be established, are not usable in prehospital practice, where the resources are scarce. To solve this issue, qSOFA score has been proposed [14] but its validity still remains under debate [38, 39]; to date, no score is validated in the prehospital setting [40, 41]. More recently, biomarker addition to scores was proposed to improve efficiency. Lactatemia is validated [42] and recognized as useful biomarker for sepsis severity and risk of mortality assessment [43], despite lactate point of care testing validity is, to date, not widely available easily in the out-of-hospital setting. Base excess and bicarbonate plasma level appears to be alternatives to lactate [44], by reflecting tissue hypoperfusion, but have not been evaluated in the prehospital setting.

Because the ROX index is a simple clinical tool, obtained in real time, easily, noninvasively measurable clinically or with a simple monitoring system, it appears to be helpful for physicians’ daily practices. Although the ROX threshold varies according to study populations [23, 26, 45], it appears that a higher value is associated with a worse prognosis. The ROX index evaluation presents the advantage not being influenced to subjectivity contrary to skin mottling score and capillary refill time assessment [46]. However, currently respiratory rate evaluation is not accurate, especially for the less sick patients [47] and could limit the ROX index use in daily practice. To improve respiratory rate accuracy measurement, devices allowing a continuous measurement [48], and smartphone applications were developed and are now available for in and out-of-hospital practice [49].

### Limitations

This study presents limitations. First, this is a retrospective analysis. Second, bias from misclassification of covariates might exist, because data were manually extracted from prehospital and in-hospital medical reports. Third, the statistical analysis does not allow any conclusion on causality. Fourth, this study focused only patients with shock, not all patients with sepsis. Finally, we only assessed the association between 30-day mortality and the first ROX index measured after MICU contact before any oxygen supplementation and did not evaluate the dynamic change in the ROX index.

Beyond all these limitations, the ROX index appears to be useful since the prehospital setting to, earlier, screen septic shock patients with a higher risk of poorer outcome.

## Conclusions

Among septic shock patients cared for by a prehospital MICU, a significant association between ROX index and 30-day mortality exists. A 1.5-fold 30-day mortality increase is observed when the prehospital ROX is lower or equal than 10. Further prospective studies are needed to confirm these preliminary results and evaluate the ability of prehospital ROX to predict sepsis outcome since the prehospital setting.

## Data Availability

Data will be made available on reasonable request.

## Abbreviations

SS: Septic shock
MICU: Mobile intensive care unit
aHR: Adjusted hazard ratio
ED: Emergency department
ICU: Intensive care unit
SAMU: Urgent Medical Aid Service
SMUR: Mobile Emergency and Resuscitation Service
SBP: Systolic blood pressure
DBP: Diastolic blood pressure
MAP: Mean arterial pressure
HR: Heart rate
SpO_2_: Pulse oximetry
RR: Respiratory rate
GCS: Glasgow coma scale
LOS: Length of stay
SOFA: Sequential Organ Failure Assessment
qSOFA: Quick Sequential Organ Failure Assessment
SAPS-2: Simplified Acute Physiology Score
FiO_2_: Inspired oxygen concentration
BMI: Body mass index
